# Comparison of soil bacterial communities in a natural hardwood forest and coniferous plantations in perhumid subtropical low mountains

**DOI:** 10.1186/s40529-014-0050-x

**Published:** 2014-06-07

**Authors:** Yu-Te Lin, Hsueh-Wen Hu, William B Whitman, David C Coleman, Chih-Yu Chiu

**Affiliations:** 1 Biodiversity Research Center, Academia Sinica, Nankang, Taipei 11529 Taiwan; 2 Department of Microbiology, University of Georgia, Athens, 30602-2605 GA USA; 3 Odum School of Ecology, University of Georgia, Athens, 30602-2602 GA USA

**Keywords:** Forest soils, Bacterial community, Bacterial diversity, 16S rRNA genes

## Abstract

**Background:**

The bacterial community of forest soils is influenced by environmental disturbance and/or meteorological temperature and precipitation. In this study, we investigated three bacterial communities in soils of a natural hardwood forest and two plantations of conifer, *Calocedrus formosana* and *Cryptomeria japonica*, in a perhumid, low mountain area. By comparison with our previous studies with similar temperature and/or precipitation, we aimed to elucidate how disturbance influences the bacterial community in forest soils and whether bacterial communities in similar forest types differ under different climate conditions.

**Results:**

Analysis of 16S ribosomal RNA gene clone libraries revealed that *Acidobacteria* and *Proteobacteria* were the most abundant phyla in the three forest soil communities, with similar relative abundance of various bacterial groups. However, UniFrac analysis based on phylogenetic information revealed differences of bacterial communities between natural hardwood forest and coniferous plantation soils. The diversities of bacterial communities of the replanted *Calocedrus* and *Cryptomeria* forests were higher than that in natural hardwood forest. The bacterial diversity of these three forest soil were all higher than those in the same forest types at other locations with less precipitation or with lower temperature. In addition, the distribution of some of the most abundant operational taxonomic units in the three communities differed from other forest soils, including those related to *Acidobacteria*, α-, β- and γ-*Proteobacteria*.

**Conclusions:**

Reforestation could increase the bacterial diversity. Therefore, soil bacterial communities could be shaped by the forestry management practices and climate differences in warm and humid conditions.

**Electronic supplementary material:**

The online version of this article (doi:10.1186/s40529-014-0050-x) contains supplementary material, which is available to authorized users.

## Background

Soil microbes play a key role in organic matter turnover and nutrient cycling (Xu et al. [2008]). In forest soils, the activity, diversity and structure of the microbial community are influenced by several environmental factors. For example, tree species diversity is a major factor affecting communities of soil microbes (Curlevski et al. [2010]; Liu et al. [2012]; Oh et al. [2012]). Soil properties variations also are considered to be among the important factors which affect the composition of soil bacterial communities. The composition of soil bacterial communities is also related to soil properties, including soil pH (Lauber et al. [2009]), soil C: N ratio (Högberg et al. [2007]) and moisture (Stres et al. [2008]). Some evidence also indicated that the community composition responds to regional climate changes in temperature and precipitation (Sowerby et al. [2005]; Nielsen et al. [2010]). In addition, disturbance of forestry management such as thinning alters soil characteristics (Bolat [2014]) and affects the soil microbial community structure and diversity (Chatterjee et al. [2008]; Curlevski et al. [2010]). The disturbance results in bacterial community shifts between natural and disturbed forest soils (Lin et al. [2011a]).

Our previous studies showed a greater bacterial diversity of a disturbed hardwood forest soil with annual precipitation ~4,000 mm in a low mountain region (~300 m a.s.l) at Huoshaoliao (Lin et al. [2011b]) than that in natural and secondary forest soils at Lienhuachi, with similar elevation but less precipitation (Lin et al. [2011c]). The bacterial community at Huoshaoliao is also more diverse than that in a perhumid natural *Chamaecyparis* forest at Yuanyang Lake forest soils, where is with relatively high elevation (~1,800 m a.s.l.) lower mean temperature (Lin et al. [2010]). Thus, high bacterial diversity could be due to forest disturbance or temperature and/or precipitation differences.

To elucidate the factor, such as forest disturbance or temperature and/or precipitation that might be important in soil bacterial composition, we investigated natural and disturbed forest soils with similar temperature and precipitation. We examined the diversity and composition of indigenous soil bacterial communities in a low mountain forest located at Wulai in northern Taiwan. This region features a hardwood forest and receives above 4,000 mm annual precipitation. The site is a subtropical, perhumid montane forest ecosystem with high soil acidity in a monsoonal part of Southeastern Asia. Parts of this region have been replanted by coniferous plantations. We prepared 16S rRNA gene clone libraries from these soils to identify soil organisms and to compare soil bacterial communities. The comparison of sequence data includes this study site at Wulai and those from other forests with different climate conditions, including a disturbed hardwood forest in Huoshaoliao (Lin et al. [2011b]), a natural *Chamaecyparis* forest in the Yuanyang Lake forest ecosystem (Lin et al. [2010]), and a hardwood forest and a *Calocedrus* plantation at Lienhuachi (Lin et al. [2011c]) (Table 1). The forests in Huoshaoliao and Yuanyang Lake represent a perhumid environment as Wulai, but the mean annual temperature in the Yuanyang Lake forest is lower. The mean annual temperature in Wulai and Lienhuachi forests is similar, but the Lienhuachi ecosystem has less precipitation and is relatively dry during the autumn and winter. The hardwood forest in Wulai and the *Chamaecyparis* forest in Yuanyang Lake have not experienced human disturbance. The Huoshaoliao forest has undergone timber harvesting for coal-mining construction and for firewood (Lin et al. [2011b]) for the heaviest disturbance. Other forest soil communities, including those in *Calocedrus* and *Cryptomeria* forests at Wulai and *Calocedrus* forest at Lienhuachi, have undergone mild disturbance. We used similar data-collection methods at all sites. We hypothesized that 1) disturbance by forest management could result in differences in composition and diversity of bacterial communities between natural hardwood and coniferous forest soils; and 2) a warm and humid climate could result in differences in bacterial communities between similar forest types with different climate conditions. We aimed to elucidate the effects of disturbance and climate conditions on the structure and diversity of communities in subtropical forest soils to help unravel the influence of forest management and climate conditions on forest soil bacterial communities.

**Table 1:** **Description of the bacterial 16S rRNA gene clone libraries of forest soils used in the study**

Location	Description of the forest	MAT^a^	Precipitation (mm)	Reference
Wulai	Natural, low mountain hardwood forest in northern Taiwan	21.0	>4,000	This study
	Low mountain *Calocedrus* plantation in northern Taiwan	21.0	>4,000	This study
	Low mountain *Cryptomeria* plantation in northern Taiwan	21.0	>4,000	This study
Huoshaoliao	Disturbed, low mountain hardwood forest in northern Taiwan	21.0	>4,000	Lin et al. ([2011b])
Lienhuachi	Natural, low mountain hardwood forest in central Taiwan	20.8	2,200	Lin et al. ([2011c])
	Low mountain *Calocedrus* plantation in central Taiwan	20.8	2,200	Lin et al. ([2011c])
Yuanyang Lake	Natural, middle altitudinal *Chamaecyparis* forest in northeastern Taiwan	12.0	>4,000	Lin et al. ([2010])

^a^MAT, mean annual temperature.

## Methods

### Site description and soil sampling

This study was conducted at Wulai, a subtropical low mountain area in northern Taiwan (24°49’ N, 118°49’ E) located near the Tonghou Stream, one of the major upstream water resources for Taipei city. This region is humid all year, with mean annual precipitation of >4,000 mm. The elevation is ~500 m a.s.l. and the mean annual temperature 21°C. The Wulai area is covered mainly by evergreen hardwood forest. Parts of the region have been replanted with coniferous plantations, including *Calocedrus formosana* and *Cryptomeria japonica* forests which are around 20 m height and have planted for more than 40 years. The soils are acidic, and the pH values of the surface soils range from 3.8 to 4.0 (Table 2). The highest cation exchange capacity (CEC) value was in the hardwood soils, which was associated with relatively high organic C and clay contents. The low base saturation in these forest soils was resulted from strong leaching of high precipitation.

**Table 2:** **Soil chemical and physical properties of study sites**

Property	Hardwood	***Calocedrus***	***Cryptomeria***
pH	4.0	3.8	3.9
Organic C (g kg^-1^)	61.5	60.8	52.7
Total N (g kg^-1^)	5.0	5.1	4.4
C/N	12.3	12.0	12.0
CEC^a^ (cmol(+) kg^-1^)	26.0	23.2	22.5
Base saturation (%)	2.4	3.2	11.6
Sand (%)	3.2	5.1	6.6
Silt (%)	43.5	45.3	45.0
Clay (%)	53.3	49.7	48.4
Soil group	Dystrudept	Dystrudept	Dystrudept

^a^Cation exchange capacity.

Soil samples were collected from hardwood, *Calocedrus* and *Cryptomeria* forests in November 2011. Broad-leaved bushes and trees scatter under the hardwood and *Calocedrus* canopy, with almost no trees under the *Cryptomeria* canopy because of the deep shade. Three replicate 50 x 50 m plots were selected for each forest type. Each replicate was separated by at least 50 m. The soils were collected with use of a soil auger 8 cm in diameter and 10 cm deep. Three subsamples collected in each plot were combined. Visible detritus, such as roots and litter, was manually removed before material was passed through a 2-mm sieve. Soils were then stored at -20°C and extraction of soil community DNA was performed within 2 weeks.

### DNA extraction, PCR and 16S rRNA gene clone library construction

The 16S rRNA gene clone libraries were constructed as described (Lin et al. [2010]). DNA of the soil community was extracted by use of the PowerSoil^®^ DNA Extraction kit (MoBio Industries, Carlsbad, CA, USA). The bacterial 16S rRNA genes were amplified by PCR with the primer set 27F and 1492R (Lane [1991]). After 15 cycles, the PCR products were cloned by use of the TOPO TA cloning kit (Invitrogen, Carlsbad, CA, USA) and the pCR2.1 vector. White colonies on selective Luria-Bertani (LB) agar plates were separated into 96-well blocks containing 1 ml LB broth plus kanamycin (50 μg ml^-1^) and grown overnight. Sterile glycerol was added to a final concentration of 10%, and an aliquot was transferred to a 96-well sequencing block. Both the sequencing and original culture blocks were stored at -80°C.

### DNA sequencing and taxonomic assessment

Bacterial clones were partially sequenced with use of the primer 27F. Sequence analysis involved use of an ABI PRISM Big Dye Terminator cycle sequencing-ready reaction kit and an ABI 3730 Genetic Analyzer (both Applied Biosystems, Foster City, CA, USA). Sequences were analyzed by use of the Mallard and Pintail programs to test for chimeras (Ashelford et al. [2005, 2006]). Sequences were taxonomically assigned by use of the naïve Bayesian rRNA classifier, with confidence threshold 80% (Wang et al. [2007]) in the Ribosomal Database Project (http://rdp.cme.msu.edu/index.jsp). The sequences have been deposited into GenBank under accession numbers JN168158-JN168654.

### Diversity estimates, library comparison and statistical analyses

We compared our data with that four soil bacterial communities from a hardwood forest in Huoshaoliao (with climate similar to Wulai but with higher disturbance), hardwood and *Calocedrus* forests in Lienhuachi (with less precipitation (2,200 mm) than in Wulai) and a *Chamaecyparis* forest in Yuanyang Lake forest (with perhumid (precipitation >4,000 mm) and cooler (mean annual temperature 12°C) conditions, as compared with Wulai) described previously (Table 1) (Lin et al. [2010, 2011b, c]). The soils in Huoshaoliao and Lienhuach were classified as Dystrudept (U.S. Soil Taxonomy) (Lin et al. [2011b, c]), same as soils in Wulai; while soils in Yuanyang Lake forest were Dystrochrept (Lin et al. [2010]). The soils in Yuanyang Lake forest were more acidic (pH 3.5) (Lin et al. [2010]), and pH value in Huoshaoliao and Lienhuach is 4.5 and 3.9-4.0, respectively (Lin et al. [2011b, c]). Diversity estimates, including ACE, Chao1 estimator, Shannon diversity index, and rarefaction curve were calculated for operational taxonomic units (OTUs) with evolutionary distance (D) of 0.03 (or about 97% 16S rRNA gene sequence similarity) using the DOTUR software package (Schloss and Handelsman [2005]). To analyze the distribution of abundant taxa within libraries, groups were constructed by use of DOTUR at a distance of ≤0.03. The taxonomic affiliation of the most abundant OTUs was screened by use of BLAST program in the NCBI Genbank database. These groups were then analyzed by use of the Fisher exact test (Agresti [1992]). UniFrac (Lozupone et al. [2006]) was used to compare the clone libraries on the basis of the phylogenetic information. The UniFrac Significance test option with 100 permutations was used to determine significant differences between each pair of samples on the basis of phylogenetic information. Jackknife Environment Clusters was used with the weighted algorithm (which considers relative abundance of OTUs) and the normalization step. Relationships between phylogenetic distances of bacterial communities and soil properties were assessed by Mantel tests as implemented in PRIMER v6 (Clarke and Gorley [2006]).

## Results

### Phylogenetic groups represented in the clone library

We derived about 50 to 60 clones of 16S rRNA genes from each of the three replicate samples collected for the forest soils, and the sequences from replicates of each vegetation type were then combined for further analyses. We obtained 168 clones for the hardwood forest and 168 and 161 clones for *Calocedrus* and *Cryptomeria* plantations, respectively. The sequence length determined was about 900 bp. Six clones were chimeras and were removed from the dataset. The remaining clones were classified into 10 phylogenetic groups (Table 3).

**Table 3:** **Phylotypes of clones in 16S rRNA gene libraries**

Phylogenetic group	Clone library (% of clones)		
	Hardwood	***Calocedrus***	***Cryptomeria***
*Acidobacteria*	63.7a	68.5a	59.6a
*Actinobacteria*	1.8a	0.6a	0.0a
*Bacteroidetes*	0.0a	1.2a	1.2a
*Chloroflexi*	0.0b	1.2ab	3.7a
*Firmicutes*	0.0b	1.2ab	1.9ab
*Nitrospira*	0.6a	0.0a	0.0a
*Planctomycetes*	4.8a	0.0b	1.9ab
*Proteobacteria*	23.8a	25.6a	23.0a
α-*Proteobacteria*	15.5a	17.9a	11.8a
β-*Proteobacteria*	1.8a	0.6a	2.5a
γ*-Proteobacteria*	4.8a	3.0a	3.7a
δ-*Proteobacteria*	1.8b	4.2ab	4.3ab
Unclassified *Proteobacteria*	0.0a	0.0a	0.6a
*Verrucomicrobia*	5.4ab	1.8b	6.2a
Unclassified bacteria	0.0a	0.0a	2.5a
Total clone numbers	168	168	161

Data with the same letter in each row indicates no significant difference by LSD test at *P*<0.05.

In the three soil libraries, *Acidobacteria* was the most abundant and accounted for more than half of the clones. *Proteobacteria* comprised about 25% of all clones and was the second most abundant group. α-*Proteobacteria* was the most abundant class, then γ-*Proteobacteria* and δ-*Proteobacteria*. The remaining phyla, *Actinobacteria*, *Bacteroidetes*, *Chloroflexi*, *Firmicutes*, *Nitrospira*, *Planctomycetes* and *Verrucomicrobia* all represented less than 7% of the clones.

Table 4 shows the 20 most abundant genera found in each forest community. The GP1, 2 and 3 were all the most abundant genera in three communities, though the relative abundance of GP3 in hardwood was less than 10%. Other genera all accounted for less than 5%. In the genera affiliated to α-*Proteobacteria*, including *Rhodoplanes* and *Bradyrhizobium*, did not distribute equally among three communities.

**Table 4:** **The 20 most abundant genera identified in hardwood forest and** ***Calocedrus*** **and** ***Cryptomeria*** **plantations**

Phylum	Genus	Percentage of clones		
		Hardwood	***Calocedrus***	***Cryptomeria***
*Acidobacteria*	GP1^a^	28.0	23.2	26.7
	GP2	22.6	27.4	17.4
	GP3	7.1	13.1	12.4
*Verrucomicrobia*	Subdivision3_genera_incertae_sedis	2.4	1.2	4.3
*Acidobacteria*	GP5	3.6	1.8	1.9
*Proteobacteria*	*Steroidobacter*	3.0	1.8	0.6
	*Rhodoplanes*	3.0	0.6	1.2
	*Bradyrhizobium*	0.6	2.4	1.9
*Verrucomicrobia*	*Spartobacteria* _genera_incertae_sedis	2.4	0.6	1.9
*Acidobacteria*	GP4	0.6	1.2	0.6
*Planctomycetes*	*Planctomyces*	1.2	0.0	1.2
*Proteobacteria*	*Pedomicrobium*	0.0	1.8	0.6
	*Burkholderia*	1.2	0.0	1.2
	*Aquicella*	0.0	0.6	1.2
*Acidobacteria*	GP6	0.0	0.6	0.6
*Actinobacteria*	*Aciditerrimonas*	0.6	0.6	0.0
*Actinobacteria*	*Mycobacterium*	1.2	0.0	0.0
*Chloroflexi*	*Ktedonobacter*	0.0	0.6	0.6
*Planctomycetes*	*Gemmata*	1.2	0.0	0.0
*Acidobacteria*	GP7	0.6	0.0	0.0

^a^GP, group.

The distribution of some bacterial groups in the three Wulai forest soils of hardwood forest and *Calocedrus* and *Cryptomeria* plantations differed from those at the other sites previously studied. Within *Proteobacteria*, α-*Proteobacteria* was the most abundant class in the three Wulai soil communities, but it did not predominate in the hardwood and *Chamaecyparis* forest soils in Lienhuachi and Yuanyang Lake, where the communities were dominant with γ- and β-*Proteobacteria*, respectively (Lin et al. [2010, 2011c]). In the *Calocedrus* plantation at Lienhuachi and the hardwood forest at Huoshaoliao, *Acidobacteria* were abundant in soils but represented less than 50% of clones (Lin et al. [2011b, c]).

### Diversity of soil bacterial communities

**Figure 1:**
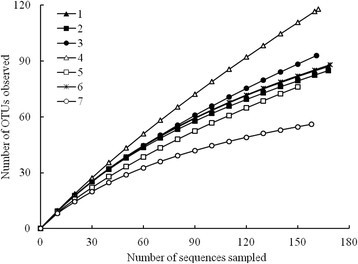
**Rarefaction curve analysis for forest soil libraries with OTUs formed at an evolutionary distance ≤0.03 (or about >97% similarity).** Site notation is as follows: (1) hardwood forest, and (2) *Calocedrus* and (3) *Cryptomeria* plantations in Wulai with warm and perhumid conditions; (4) hardwood forest in Huoshaoliao (with climate similar to Wulai but with higher disturbance); (5) hardwood forest and (6) *Calocedrus* plantation in Lienhuachi (with warmer and less precipitation conditions than in Wulai); (7) *Chamaecyparis* forest in Yuanyang Lake (with perhumid and cooler conditions, as compared with Wulai). Values for Huoshaoliao, Lienhuachi and Yuanyang Lake forests were calculated from data in Lin et al. ([2010, 2011b, c]).

Despite the similar relative abundances of various bacterial groups among the three Wulai soil communities, ACE index and Chao1 nonparametric richness estimators revealed greater diversity for the *Cryptomeria* soil community than hardwood and *Calocedrus* soil communities (Table 5). Rarefaction analysis also showed that slope of curve was steeper for the *Cryptomeria* community, which supported above conclusion (Figure 1). In addition, 42% of clones in *Cryptomeria* soils were single-member OTUs (singletons). By comparison, 29% and 30% of clones in hardwood and *Calocedrus* soils, respectively, were singletons. As well, the bacterial community was more diverse in the heavily disturbed hardwood in Huoshaoliao forest than *Cryptomeria* forest in Wulai. The diversity of bacterial communities were similar between hardwood and *Calocedrus* soils in Wulai and hardwood and *Calocedrus* soils in Lienhuachi; the *Chamaecyparis* forest soils in Yuanyang Lake was the least diverse (Figure 1).

**Table 5:** **Diversity indices for the three 16S rRNA gene clone libraries**

Index	Hardwood	***Calocedrus***	***Cryptomeria***
S^a^	87	85	93
N^b^	168	168	161
ACE	140	165	266
Richness^c^	0.28	0.29	0.42
Shannon^d^	4.21	4.20	4.24
Chao1	138	173	258
95% COI^e^	112-193	126-271	174-427

Calculations were based on operational taxonomic units formed at an evolutionary distance of <0.03 (or ~97% similarity).

^a^S, number of operational taxonomic units observed.

^b^N, number of sequences.

^c^Richness = (number of singleton OTUs-1)/logN. The maximum value is (N-1)/logN. The observed/maximum possible value is reported.

^d^Shannon diversity index (*H*).

^e^95% confidence interval for Chao1 estimator.

### Abundant OTUs in soil bacterial communities

**Figure 2:**
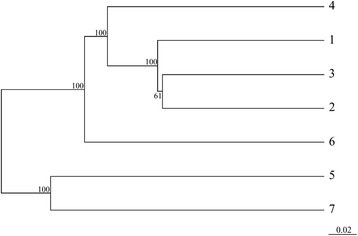
**A dendrogram from UniFrac Jackknife environment clusters analysis of 16S rRNA gene clone libraries.** Analysis involved weighted data. Numbers at nodes indicate the frequency with which nodes were supported by Jackknife analysis. The length of the scale bar indicates a distance of 0.02. Site notation is as described for Figure 1.

Analyses of community composition revealed that the three soil communities in Wulai significantly differed from each other (*P*<0.05) and from our previously studied hardwood soils in Huoshaoliao, hardwood and *Calocedrus* soils in Lienhuachi and *Chamaecyparis* soils in Yuanyang Lake (*P*<0.05) (data not shown). On cluster analysis of the clone libraries, the *Calocedrus* and *Cryptomeria* communities in Wulai were in the same cluster and differed from that of the hardwood community (Figure 2). Furthermore, the Wulai communities formed a cluster near the Huoshaoliao community, separated from that formed by hardwood and *Calocedrus* soils in Lienhuachi and the *Chamaecyparis* forest in Yuanyang Lake.

We found differences in composition by examining of the abundant OTUs with size larger than 10 (Table 6). Because representatives of each OTU were obtained from independent replicates in multiple sampling locations and the representation was similar in the different sample replicates (data not shown), their abundance was not due to PCR or cloning artifacts or to a single unusual sample. Among the three Wulai communities, these clones were mainly representative of *Acidobacteria* and α-*Proteobacteria* and the distribution differed from that in the other soil communities. The β-proteobacterial *Burkholderia*-affiliated OTU was abundant in *Chamaecyparis* soils in Yuanyang Lake but nearly absent in Wulai forest soils. Another abundant OTU closely related to the genus *Stenotrophomonas* of γ-*Proteobacteria* was abundant only in the hardwood forest in Lienhuachi and not detected in Wulai soils. Finally, the most abundant OTU in Wulai soils, acidobacterial GP1-affiliated OTU, with 23 clones, was less abundant in Huoshaoliao, Lienhuachi, and absent in Yuanyang Lake forest soils (Table 5).

**Table 6:** **Distribution of the most abundant operational taxonomic units (OTUs) in forest soil libraries** ^**a**^

Group (N^b^)	Taxonomic affiliation	Clone library of each forest (N^c^)						
		1	2	3	4^d^	5^e^	6^e^	7^f^
*Acidobacteria*								
27	*Acidobacteria* GP1^g^ (EU445214)	3a	7a	7a	3a	2ab	5a	0b
27	*Acidobacteria* GP1 (EU680424)	9a	4ab	10a	3a	1bc	0bc	0c
22	*Acidobacteria* GP3 (AY963381)	6ab	8a	3ab	2abc	1bc	2abc	0c
17	*Acidobacteria* GP1 (DQ451448)	1abc	6a	4ab	0bc	1abc	5ab	0c
17	*Acidobacteria* GP2 (EU881239)	1a	3a	3a	1ab	3a	6a	0b
15	*Acidobacteria* GP2 (EU680416)	4a	1ab	2ab	3a	1ab	4a	0b
11	*Acidobacteria* GP1 (GQ487979)	2	1	4	4	0	0	0
α-*Proteobacteria*								
30	*Stenotrophomonas* (FJ894817)	0b	0b	0b	0b	28a	2b	0b
25	*Rhodoplanes* (EU881284)	4	3	3	6	1	3	5
16	*Bradyrhizobium* (FJ592549)	1	4	4	2	0	1	4
16	*Steroidobacter* (JF833908)	2ab	3ab	0ab	3ab	4a	3ab	1b
β-*Proteobacteria*								
28	*Burkholderia* (AY949190)	1b	0b	0b	0b	2b	0b	25a

Site notation is as follows: (1) hardwood forest, and (2) *Calocedrus* and (3) *Cryptomeria* plantations in Wulai; (4) hardwood forest in Huoshaoliao; (5) hardwood forest and (6) *Calocedrus* plantation in Lienhuachi; (7) *Chamaecyparis* forest in Yuanyang Lake.

^a^OTUs formed at an evolutionary distance ≤0.03 (or about >97% similarity). Data with the same letter in each row indicates no significant difference by LSD test at *P*<0.05.

^b^Total number of clones in an OTU.

^c^Number of clones in each library.

^d^Data from Lin et al. ([2011b]).

^e^Data from Lin et al. ([2011c]).

^f^Data from Lin et al. ([2010]).

^g^GP, group.

### Correlation between community composition and soil properties

Mantel tests of a community assemblage matrix calculated from OTU abundances and pairwise environmental matrices indicated significant correlations (*P*<0.05) between community and elevation, mean annual temperature and precipitation (Table 7). Other variables, including pH, organic C, total N, C: N ratio and proportion of clay were not significantly correlated with bacterial community structure in these forest soils.

**Table 7:** **Correlation of bacterial communities with environmental properties** ^**a**^

Soil properties	Spearman rank correlation
Elevation	**0.621**
Mean annual temperature	**0.604**
Precipitation	**0.698**
pH	0.285
Organic C	0.414
Total N	0.407
C:N ratio	0.108
Clay	0.418

^a^Values represented in bold are *P* ≤ 0.05.

## Discussion

Here, we report on differences in structure among bacterial communities of three Wulai forest soils. In these three soil libraries, *Acidobacteria* and *Proteobacteria* were the major phyla, and the relative abundance of various bacterial groups was similar. However, the three libraries significantly differed in microbial composition. Soil microbial communities are different between conifer and broad-leaved forests (Ushio et al. [2008]), which could release different quality and quantity of litter and root exudates (Sauheitl et al. [2010]). Forest management could alter soil properties, microclimatic conditions, microbial biomass (Bolat [2014]), and thereby affect the functional diversity of soil microbial communities (Gömöryová et al. [2009]). The composition of the soil microbial community could be influenced by the soil C/N ratio and the response of trees to this ratio (Högberg et al. [2007]). Lucas-Borja et al. ([2012]) indicated that tree species affect the biomass of the soil microbial community and its structure, which could explain the different bacterial clusters we found between hardwood and *Calocedrus* and *Cryptomeria* soils in Wulai. In addition, interactions between trees and understory plants in the *Calocedrus* plantation could also structure a different community from that in *Cryptomeria* soils (Mitchell et al. [2012]). Differences in climate conditions could also structure the composition of the bacterial community. A landscape-scale study in Scotland showed that the composition of soil bacterial community was related to variation in precipitation (Nielsen et al. [2010]). Precipitation could affect bacterial communities indirectly by changing the soil moisture, which may have resulted in composition differences between Wulai and Lienhuachi forest communities (Guenet et al. [2012]). Soil pH is a well-known factor to predict bacterial diversity and structure (Tripathi et al. [2012]). Some other soil properties, including organic C, total N and C/N ratio, also affect the relative abundance of copiotrophic/oligotrophic microbes (Nemergut et al. [2010]), though they are all not significantly correlated with soil communities in this study. Environmental factors caused by climate condition differences, as well as management, could have important effects on bacterial communities.

Rarefaction analysis revealed all that three Wulai forest soil communities were more diverse than natural *Chamaecyparis* soil communities in Yuanyang Lake forest. The disturbance of forest soils could increase the diversity of microbial communities (Jangid et al. [2010]; Lin et al. [2011a]) and result in increased bacterial diversity in the *Cryptomeria* soils in Wulai. The most disturbed soil bacterial community of Huoshaoliao forest with similar climate conditions to that for Wulai communities, showed a highly diverse bacterial community.

Besides disturbance, temperature might cause the low diversity in the *Chamaecyparis* forest community. Temperature and moisture condition changes have been linked to changes in microbial community composition (Lipson [2007]). The relatively high altitude of the *Chamaecyparis* forest in Yuanyang Lake implies increased environmental harshness, including lower annual temperature, which in turn affects the composition and diversity of the soil bacterial community (Lipson [2007]). The bacterial diversity among different forest types, including hardwood and *Calocedrus* forests in Wulai and Lienhuachi, was similar, despite different precipitation conditions. Brockett et al. ([2012]) found soil moisture to be the major factor influencing microbial community structure across seven biogeoclimatic zones in western Canada. Data for more forests with different degree of disturbance and/or precipitation are needed to investigate whether disturbance and precipitation are a major factor influencing bacterial community diversity.

The phylum *Acidobacteria* is distributed widely in various soil environments (Zimmermann et al. [2005]; Araujo et al. [2012]; Meng et al. [2013]), and its abundance is significantly correlated with soil pH (Lauber et al. [2009]). The forests we compared all possessed low soil pH values, and *Acidobacteria* was also abundant in most of the microbial communities. However, its relative abundance was lower in the hardwood forest soils in Lienhuachi and *Chamaecyparis* soils in Yuanyang Lake than in other communities. In a continental scale study, *Acidobacteria* accounted for more than 60% of the microbial community (Lauber et al. [2009]). The species are also known to be cultured in media with low pH (Sait et al. [2006]). Including data of soil bacterial communities in different forest type would be worthwhile to elucidate which factors influence the abundance of *Acidobacteria* in these two communities with low soil pH. A recent study of the whole-genome sequences of three *Acidobacteria* strains revealed that they are versatile heterotrophs and have slow metabolic rates under low nutrient conditions (Ward et al. [2009]). In this study, however, the abundant genera of this phylum, including GP1, 2, and 3, were not closely related to the described strains. Thus, determining the functional and physical roles in the ecosystems is difficult. In light of the abundance of the phylum in different forest soil communities, further examination of clones in pure cultures for physiological characteristics should have high priority.

*Proteobacteria* are abundant in different forest soil communities (Janssen [2006]). The class α-*Proteobacteria* represented most of the proteobacterial clones in our three Wulai communities. The clones related to the order *Rhodoplanes* and *Bradyrhizobium* were found in these three communities. They include species involved in N fixation, organic matter decomposition, and plant growth promotion (Zhang and Xu [2008]; Yarwood et al. [2009]). Their physiological properties suggest the potential role of the bacterial community for N budget and nutrient cycles in these ecosystems.

## Conclusion

In conclusion, plantation changes bacterial composition between natural hardwood forest and coniferous plantations. The climate differences in warm and humid conditions could determine difference of community structure, as well as disturbance of forestry management practices. The forest management could also increase the bacterial diversity. These findings could help improve our understanding of variations in soil bacterial communities between natural and disturbed forests under different climate conditions. Also, related information could be applied to conservation efforts in the fields of forest management and climate change.

## Authors’ original submitted files for images

Below are the links to the authors’ original submitted files for images. Authors’ original file for figure 1 Authors’ original file for figure 2
