# Supplementary figures and images for: Comparison of soil bacterial communities in a natural hardwood forest and coniferous plantations in perhumid subtropical low mountains
Source: Bot Stud. 2014 Jun 7;55:50. doi: 10.1186/s40529-014-0050-x (PMC5432764; doi:10.1186/s40529-014-0050-x)

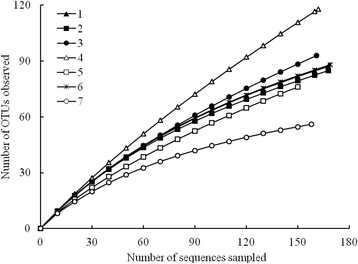

Supplement: Supplementary file 1 — Authors’ original file for figure 1 [file 40529_2014_50_MOESM1_ESM.gif]

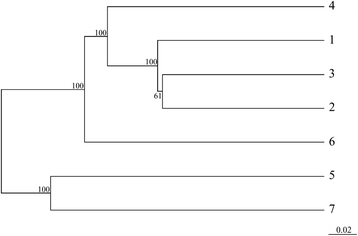

Supplement: Supplementary file 2 — Authors’ original file for figure 2 [file 40529_2014_50_MOESM2_ESM.gif]
